# Expression of Concern: Targeted plasmalogen supplementation: effects on blood plasmalogens, oxidative stress biomarkers, cognition, and mobility in cognitively impaired persons

**DOI:** 10.3389/fcell.2026.1796393

**Published:** 2026-01-30

**Authors:** Frontiers Editorial Office

With this notice, Frontiers states its awareness of serious concerns regarding compliance with local legislation in relation to the article “Targeted Plasmalogen Supplementation: Effects on Blood Plasmalogens, Oxidative Stress Biomarkers, Cognition, and Mobility in Cognitively Impaired Persons” published on 6th July 2022. Our Research Integrity team is conducting an investigation in full accordance with our procedures. The situation will be updated as soon as the investigation is complete.

**UPDATE:** This article has now been retracted. Please find the full retraction here: https://doi.org/10.3389/fcell.2026.1964604

